# Solanales Stem Biomechanical Properties Are Primarily Determined by Morphology Rather Than Internal Structural Anatomy and Cell Wall Composition

**DOI:** 10.3390/plants9060678

**Published:** 2020-05-27

**Authors:** Ilana Shtein, Alex Koyfman, Amnon Schwartz, Zoë A. Popper, Benny Bar-On

**Affiliations:** 1 Eastern R&D Center, Ariel 4077625, Israel; ilanash@ariel.ac.il; 2 Department of Mechanical Engineering, Ben-Gurion University of the Negev, Beer-Sheva 84105, Israel; alexko88@gmail.com; 3 Nuclear Research Center-Negev, O. Box 9001, Beer-Sheva 84190, Israel; 4 The Robert H. Smith Faculty of Agriculture, Food & Environment, The Robert H. Smith Institute of Plant Sciences and Genetics in Agriculture, The Hebrew University of Jerusalem, Rehovot 7610001, Israel; amnon.schwartz@mail.huji.ac.il; 5 Botany and Plant Science, Ryan Institute for Environmental, Marine and Energy Research, School of Natural Sciences, National University of Ireland Galway, H91 TK33 Galway, Ireland; zoe.popper@nuigalway.ie

**Keywords:** climbers, immunohistochemistry, cell walls, evolution, effective modulus, stem stiffness

## Abstract

Self-supporting plants and climbers exhibit differences in their structural and biomechanical properties. We hypothesized that such fundamental differences originate at the level of the material properties. In this study, we compared three non-woody members of the Solanales exhibiting different growth habits: (1) a self-supporting plant (potato, *Solanum tuberosum*), (2) a trailing plant (sweet potato, *Ipomoea batatas*), and (3) a twining climber (morning glory, *Ipomoea tricolor*). The mechanical properties investigated by materials analyses were combined with structural, biochemical, and immunohistochemical analyses. Generally, the plants exhibited large morphological differences, but possessed relatively similar anatomy and cell wall composition. The cell walls were primarily composed of hemicelluloses (~60%), with α-cellulose and pectins constituting ~25% and 5–8%, respectively. Immunohistochemistry of specific cell wall components suggested only minor variation in the occurrence and localization between the species, although some differences in hemicellulose distribution were observed. According to tensile and flexural tests, potato stems were the stiffest by a significant amount and the morning glory stems were the most compliant and showed differences in two- and three-orders of magnitude; the differences between their effective Young’s (Elastic) modulus values (geometry-independent parameter), on the other hand, were substantially lower (at the same order of magnitude) and sometimes not even significantly different. Therefore, although variability exists in the internal anatomy and cell wall composition between the different species, the largest differences were seen in the morphology, which appears to be the primary determinant of biomechanical function. Although this does not exclude the possibility of different mechanisms in other plant groups, there is apparently less constraint to modifying stem morphology than anatomy and cell wall composition within the Solanales.

## 1. Introduction

“Plants become climbers/…/to reach light, and to expose a large surface of leaves to its action and to that of the free air. This is effected by climbers with wonderfully little expenditure of organized matter, in comparison with trees, which have to support a load of heavy branches by a massive trunk” [1].

The majority of vascular plants possess an erect habit with free-standing, self-supporting stems [2,3,4]. Climbers differ from erect plants in that their growth strategy depends on firm external supports; they could be called structural parasites [5]. Simple climbers are scramblers, they lean on other plants for support, while more sophisticated twining plants coil around the supporting host. Mechanical support and hydraulic supply are two basic and structurally conflicting functional requirements of the stem architecture [6]. Mechanical support is generally provided by thick-walled fiber cells and narrow xylem vessels, while water most efficiently flows through wide and abundant xylem vessels that are mechanically weak. In self-supporting stems, these functions are in conflict. Climbing plants have limited the necessity for mechanical support, and tend to possess narrow stems with extremely large, hydraulically efficient xylem vessels [7,8,9,10,11]. There is evidence of further global trends in the structure of climbers, including increased leaf biomass, faster growth rate, deeper roots, and relatively thin and nutrient-rich leaves ([12] and references therein). Furthermore, self-supporting plants and climbers possess completely different biomechanical properties at different stages of their development. Juvenile unsupported parts of climber stems were shown to be relatively rigid and to have a higher Young’s modulus compared to those of older stems, while self-supporting plants exhibited opposite trends with their juvenile stems tending to be less rigid and having a lower Young’s modulus than more mature stems [13,14,15]. Such fundamentally different mechanical properties of climber stems could result from differences at the whole-plant-, tissue-, and cell wall-levels.

Plant cell walls are complex materials, comprised of load bearing cellulose microfibrils, embedded in a soft matrix of pectins, hemicelluloses, and various glycoproteins [16]. Cell wall composition changes in response to different developmental, environmental, and spatial contexts [17]. Each cell wall component group is complex and many undergo further modification. For example, homogalacturonans are typically synthesized in esterified form and are later deesterified as the cells/tissues age. In addition, wall components interact with each other, and therefore influence the functional properties of cell walls [18,19,20,21]. The cell wall composition of broad taxonomic groups is relatively well known [22,23,24,25,26,27], although the spatial organization, and interactions between many of the cell wall components is poorly understood. 

To date, relatively little research has been conducted on the cell wall properties of climbing plants. [28,29] found that gelatinous fibers (G-fibers) are present in stems and tendrils of many species of twining and coiling vines. Gelatinous fibers often have a mechanical function, and are structurally different by having an inner gelatinous, cellulose-rich cell wall layer (G-layer) [30]. The G-fibers in vines were shown to contain mucilaginous pectins and arabinogalactan proteins (AGPs) in their G-layer [28,29]. Tendrils and stems of vines are thought to elongate maximally, with G-fibers causing tension, thereby inducing coiling. 

Another study, investigating the cell wall structure in two tropical liana species (*Condylocarpon guianense* and *Bauhinia guianensis*), that switch from a self-supporting to a non-self-supporting growth phase on establishing contact with a support, found that each growth phase is associated with a specific anatomy; the self-supporting phase is stiff and has narrow vessels and small wood rays, and the non-self-supporting stems being more flexible and has wide vessels and broad wood rays [31]. Although liana wood types were found to have a similar monosaccharide profile to hardwoods, the ontogenetic differences in stiffness and anatomy correlated with specific changes in cell wall composition including; increased hemicellulose content of flexible wood compared to stiff wood, lignin composition (syringyl: guaiacyl unit ratios), and changes in the cellulose microfibril angle [31]. However, this work indicates that there is uncertainty surrounding the significance of the differences in cellulose microfibril angle, as significantly larger angles were associated with flexible wood as compared to stiff wood in *Condylocarpon* (44° compared to 30°), with contrastingly smaller angles found in flexible wood in *Bauhinia* (6° compared to 11°). Since the microfibril angle is thought to be significant only for mature woody tissues and tissues also become lignified at maturity [31], we elected to focus our studies on stems at an earlier, immature, developmental stages i.e., unlignified and non-woody with the aim of elucidating any changes in hemicellulose composition and/or distribution related to growth habit. 

In this study, we used biomechanical analyses combined with anatomical, immunocytochemical, and biochemical approaches to compare three non-woody members of the Solanales with different growth habits: a self-supporting plant (potato, *Solanum tuberosum*), a twining climber (morning glory, *Ipomoea tricolor*) and a trailing plant (sweet potato, *Ipomoea batatas*). Solanaceae and Convolvulaceae are two large, closely related families, belonging to the order Solanales and diverged from their common ancestor ~70 million years ago [32,33]. Both families include many structural and growth forms. They also share many similarities, including their secondary metabolite profiles and anatomical structure [33]. We compared the young mature parts of the stem i.e., stems were at developmentally comparable stages and had the characters of the mature stem of that species e.g., the vine (*Ipomea tricolor*) stem was no longer in the searching stage. By studying related species, with different growth habits, we aimed to determine whether tissue structure and cell wall composition influence the stems’ biomechanical properties.

## 2. Materials and Methods

### 2.1. Plant Material

We investigated the stem properties in three members of the order Solanales. Each species had a different growth habit and stem type: (1) Potato (*Solanum tuberosum* cv. Nicola, Solanaceae), an erect, self-supporting plant, (2) Sweet potato (*Ipomea batatas* cv. Georgia jet, Convolvulaceae), a trailing plant, and (3), Morning glory (*Ipomea tricolor*, Convolvulaceae), a climbing plant. 

Virus-free *Solanum tuberosum* ‘Nicola’ tubers and *Ipomoea batatas* ‘Georgia Jet’ cuttings were kindly provided by the Israeli Agricultural Extension Services [34]. It is important to note that while some sweet potato varieties are grown vertically, they are actually tied to the supports, as they are not vines and are unable to climb and attach themselves to a support (Z. Dar, personal communication). The variety used in the current study (‘Georgia Jet’), like other sweet potato cultivars is a creeper, and is typically grown horizontally. *Ipomoea tricolor* plants were obtained from a commercial nursery (Givat Brenner Nursery, Givat Brenner Kibbutz, Israel). The plants were grown in 10 L pots with four plants per pot. *Ipomoea tricolor* plants were allowed to twine around thin bamboo poles.

The plants were grown in a greenhouse (Phytotron) at the Faculty of Agriculture, Rehovot, Israel). Potatoes were grown under short day conditions, at 22 °C. Sweet potatoes and morning glory were grown under long days, at 28 °C. Sensors installed in each room monitored the conditions and were controlled by a computerized system. The VPD range was 1.6–1.9 kPa in both chambers.

#### Sampling

For the three species studied, only mature stem parts were examined. Stems were sampled after 1.5 months of growth. The uppermost mature internode was examined in all experiments for all species. For *I. tricolor* plants special care was taken to sample only internodes that were fully twined around a pole, and thus were certainly completely out of their self-supporting phase. For biomechanical measurements, entirely cut stems were immediately placed in wet paper towels inside plastic bags until the measurements were carried out.

### 2.2. Biomechanical Measurements

To identify the macro-mechanical characteristics of the stems, comparative tensile testing and 3-point-bending experiments were performed using a universal testing machine (Instron 5965) [35,36]. Cut stems were transferred to the lab in wet paper towels inside a plastic bag, and transferred to buckets full of water till the measurements. To prevent major loss of turgor, stem segments were tested within 5 min of excision. During both tensile and 3-point-bending tests, a progressive displacement was applied to the point of action and the corresponding reaction force was recorded. A displacement rate of 10 mm/min was used for the tensile experiments, and a rate of 1 mm/min was used to the bending experiments. The stems are non-woody, and thus are difficult to hold in place in the machine; yet, no slipping effects were observed along the experiments and clean linear curves for the stiffness calculations were obtained. For tensile experiments, the edges of the samples were coated with adhesive tape to prevent stress concentrations. For 3-point-bending experiments, the spans between supports were empirically determined by preliminary experiments for each species. The spans were set as 80 mm in *S. tuberosum* and 30 mm in both *I. tricolor* and *I. batatas*. The span-to-depth ratio (SDR) of the *I. batatas* specimens ranged from 7.3–12, from 12.7–18.8 for the *I. tricolor* specimens, and, 13.3–21.1 for the *S. tuberosum* specimens. These SDR values were found to be sufficient to generate a classical beam bending deflection (i.e., negligible shear effects) in the 3-point-bending experiments (see Supporting Information) [37]. Number of samples was *N* = 10 (*I. batatas*), *N* = 10 (*S. tuberosum*), *N* = 6 (*I. tricolor*) for the tensile tests; *N* = 9 (*I. batatas*), *N* = 7 (*S. tuberosum*), *N* = 7 (*I. tricolor*) for the bending tests.

**Figure 1:**
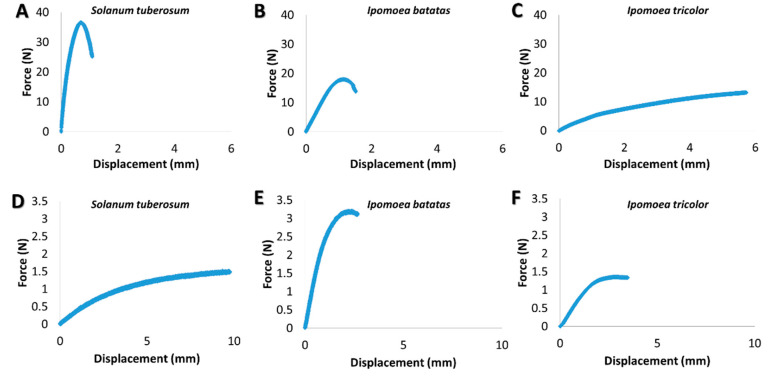
Experimental force-displacement curves of biomechanical measurements of the stems. (**A**–**C**) Tensile tests, (**D**–**F**) 3-poin-bending tests. (**A**,**D**) *S. tuberosum*, (**B**,**E**) *I. batatas*, (**C**,**F**) *I.tricolor*.

Selected experimental force-displacement curves from the tensile and bending experiments are shown in Figure 1. The tensile stiffness ($k_{T}$) and the bending stiffness ($k_{B}$) parameters are calculated from the initial slopes of the force-displacement curves (tensile and bending, respectively). Since both stiffness parameters are structural characteristics, they typically scaled with the sample length via $k_{T}\propto 1/L$ and $k_{B}\propto 1/L^{3}$ [38]. To account for the differences in specimen lengths and to provide an adequate comparison in their stiffness parameters, calibrated tensile, and bending stiffness parameters ($K_{T}$ and $K_{B}$) were calculated for a uniform reference specimen length for each experiment type ($L_{T}=53\;\mathrm{mm}$ for tensile and $L_{B}=30\;\mathrm{mm}$ for bending) via:

(1) $$K_{T}=k_{T}\times \left(L/L_{T}\right)$$

(2) $$K_{B}=k_{B}\times {\left(L/L_{B}\right)}^{3}$$

Next, the effective tensile modulus ($E_{T}$) and bending modulus ($E_{B}$), of the samples were calculated via classical relations [39]:

(3) $$E_{T}=k_{T}\times \frac{L}{A}$$

(4) $$E_{B}=\frac{1}{48}\times k_{B}\cdot \frac{L^{3}}{I}$$

where $A=\pi /4\times D^{2}$ and $I=\pi /64\times D^{4}$ are the cross-sectional area and second moment of area, respectively, D is diameter. Note that since each of these moduli parameters is an effective material property, i.e., not a structural property, it can be directly used for comparison without a prior calibration (unlike the stiffness parameters).

### 2.3. Anatomy

Prior to the bio-mechanical testing, morphological parameters of the stem segments, i.e., internode length and diameter, were measured. From these parameters, the slenderness ratio was calculated as:

(5) $$Slenderness\;ratio=\frac{Internode\;length}{Internode\;diameter}$$

For histology, 0.5 mm long pieces of mid-internode were fixed in FAA (5:5:90, formalin: acetic acid: 70% ethanol *v*/*v*/*v*) immediately after sampling. FAA-fixed samples were embedded in resin. For resin embedding (Hisoresin^®^ kit, Leica, Wetzlar, Germany) the samples were dehydrated in a graded ethanol series (70–95%), transferred to the infiltration solution for 48 h at 10 °C, transferred to molds with embedding medium, and polymerized for 1 week at −4 °C [40]. The embedding media was then allowed to polymerize at room temperature. After the embedding mixture had become viscous, the polymerization process was completed at 40 °C [41]. Cross sections were cut using a rotary microtome (Leica, Wetzlar, Germany) and stained with Toluidine Blue O [42]. The sections were viewed and photographed under a stereo microscope (Olympus SZ2-ILST) equipped with a camera (Olympus LC20). Phloroglucinol-HCl staining for lignin was carried out as described by [11] and sections observed as before. 

### 2.4. Image Analysis

Image analysis was carried out using ImageJ software [43].

From the micrographs stem area (A) and perimeter (P) as measured in ImageJ, circularity (a measure of how closely the shape approaches a perfect circle) was calculated as:

(6) $$C=4\pi \times \frac{A}{P^{2}}.$$

### 2.5. Immunohistochemistry

Immunolocalization was carried out on resin embedded sections [44,45]. All the experiments were carried out in triplicate. Monoclonal antibodies (mAbs) with affinities for epitopes present in specific cell wall components were used to identify and map specific cell wall components within the cell walls (Table 1). 

The sections were blocked with MP/PBS (PBS (phosphate-buffered saline) containing MP (3% *w*/*v* milk protein)) for 30 min, washed in PBS and incubated with mAbs for 1 h. Sections were then washed in PBS and incubated for 1.5 h with primary monoclonal antibodies diluted in MP/PBS at a ratio of 1:5. The controls were incubated in MP/PBS without the primary antibody. Subsequently, the sections were washed several times with PBS and incubated with secondary antibody (either rat or mouse, see Table 1) diluted 1:100 in MP/PBS in dark for 1 h. Sections were washed with PBS, stained with 0.25 mg mL^−1^ calcofluor white (Sigma, St Louis, MO, USA) and mounted in anti-fade agent (Citifluor AF2, Agar Scientific, Stansted, UK). The sections were then viewed using an epifluorescence Olympus IX51 microscope, equipped with a FITC filter (Fluorescence- x-cite series 120, EXFO), and captured using an Olympus DP71 digital camera and Olympus Cell Sens software. 

Additionally, prior to immunolabeling with mAbs that recognize and bind to hemicelluloses the plant, sections were also pre-treated with pectate lyase. This pre-treatment removes pectins. Pectins have been reported to mask the labeling of other cell wall components, including hemicelluloses such as xyloglucans [49].

### 2.6. Fractionation of Cell Wall Polymers into Broad Classes

For cell wall fractionation, whole internodes were placed in ethanol 70% (*v*/*v*) immediately after sampling and stored until use. 

#### 2.6.1. Preparation of Alcohol-Insoluble Residue

Internodes preserved in 70% (*v*/*v*) ethanol were washed in distilled water and homogenized using a mortar and pestle. The samples were stirred in 70% (*v*/*v*) ethanol (acidified with 1% (*v*/*v*) formic acid), and centrifuged. The pellet was washed several times in 70% (*v*/*v*) ethanol and centrifuged. The resulting cell wall-rich alcohol-insoluble residue (AIR) was washed in acetone, and completely dried under the fume hood. 

#### 2.6.2. Separation of Polysaccharides

AIR was stirred in phenol: acetic acid: water (2:1:1, *w*/*v*/*v*) at 70 °C for 1 h, filtered, washed in ethanol to remove proteins, and centrifuged. Subsequently, the cell wall polysaccharides were separated into six fractions: ‘pectin I’, ‘pectin II’, ‘hemicellulose a’, ‘hemicellulose b’, ‘α-cellulose’, ‘wash’ as described by [54]. In short, the material was twice incubated in ammonium oxalate to separate the ‘pectin I’ and ‘pectin II’ fractions, incubated in NaOH, neutralized with acetic acid and separated into soluble (‘hemicellulose b’) and insoluble (‘hemicellulose a’) fractions. The remaining pellet was washed and from it the insoluble α-cellulose was obtained, while the pooled washings were ‘wash’ fraction. The fractions were dialyzed, freeze-dried, and weighed. 

### 2.7. Statistical Analysis

Analysis of variance (ANOVA) (JMP Pro 11 Statistical Software; SAS Institute Inc., Cary, NC, USA) was used to determine differences between treatments at *p* ≤ 0.05. Tukey-Kramer post-hoc means comparisons were made to compare the significantly different treatments. 

## 3. Results

### 3.1. Structural Differences between the Stem Types

**Figure 2:**
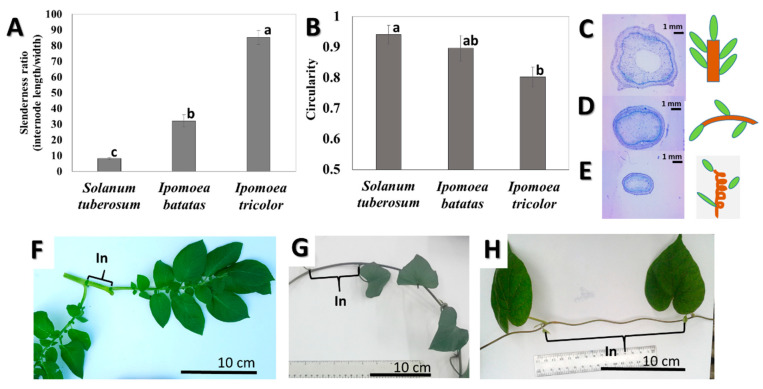
Stem morphology of the self-supporting potato (*Solanum tuberosum*) plant, trailing sweet potato (*Ipomoea batatas*) plant and twining climber morning glory (*Ipomoea tricolor*): Slenderness ratio (**A**,**F**–**H**), circularity (**B**–**E**), comparative cross-sectional and longitudinal stem profiles of (**C**,**F**) potato, (**D**,**G**) sweet potato, (**E**,**H**) morning glory. *N* = 6. *p* values were *p* ≤ 0.0001 for slenderness ratio and *p* ≤ 0.0257 for circularity. Different letters above bars annotate significant difference according to the Tukey-Kramer post-hoc test. Note that in (**H**) the climber stem is shown after uncoiling, it was initially completely coiled around the support.

We studied three related herbaceous species, belonging to the order Solanales, each having a different stem type. Potato (*Solanum tuberosum*) is an erect plant, whereas sweet potato (*Ipomoea batatas*) is a trailing plant and morning glory (*Ipomoea tricolor*) is a twining climber. We investigated the characteristics of the uppermost mature internodes of all three species in order to ensure that developmentally comparable stems were studied. The morphological structure of the different stem types was indeed found to be different. The slenderness ratio (the ratio between node length and width) was significantly the lowest in the erect stems of potato, intermediate in sweet potato, while the twining stems of morning glory were the most slender (Figure 2A,F–H). In cross-section, potato and sweet potato stems were relatively circular, while stems of the climber, morning glory, were both very slender and ellipsoid (Figure 2B,C–E).

**Figure 3:**
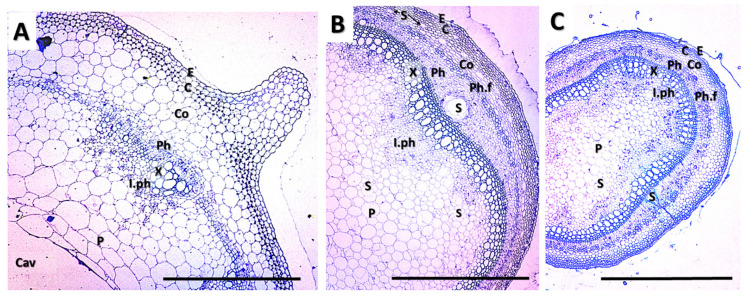
Anatomy of the stem in (**A**) potato (*Solanum tuberosum*), an erect plant, (**B**) sweet potato (*Ipomoea batatas*), trailer, and (**C**) morning glory (*Ipomoea tricolor*), a twining climber visualized by staining with toluidine blue (TBO). E—epidermis, C—collenchyma, Co—cortex, Ph—phloem, Ph. f.—phloem fibers, I. ph.—inner phloem, S—secretory cavity, P—pith, Cav—empty cavity. Scale bar—1 mm.

The anatomical structure of the uppermost mature internodes in all three species was somewhat similar. The stems are herbaceous, mostly unlignified (Supplementary Figure S1) with bicollateral vascular bundles (having both inner and outer phloem) and collenchyma at the periphery (Figure 3), with similar collenchyma proportions present in all three species ranging between 8% to 10% of the total stem area. A large pith comprised around 45% of the stem area in *S. tuberosum* and *I. batatas*, and 39% in *I. tricolor*. However, there were also certain structural differences between the species. Only the potato stems were observed to have an empty pith (Figure 2C and Figure 3A). The periphery of the potato stem is strengthened by large thick-walled collenchyma cells with typical thick cell corners that are more pronounced than observed for the other two species investigated. Vascular tissues in potato are constricted to separate small vascular bundles, and comprise only about 9% of the total stem area. In both *Ipomoea* species, the vascular tissue is continuous around the stem and is comprised of numerous xylem vessels (Figure 3B,C)—the xylem comprises around 18% of the stem area in both species. In addition, there are secretory cavities (laticifers) present both in the pith and in the cortex. On wounding, *I. batatas* stems often exclude milky fluid (latex). 

### 3.2. Biomechanical Stem Parameters

**Figure 4:**
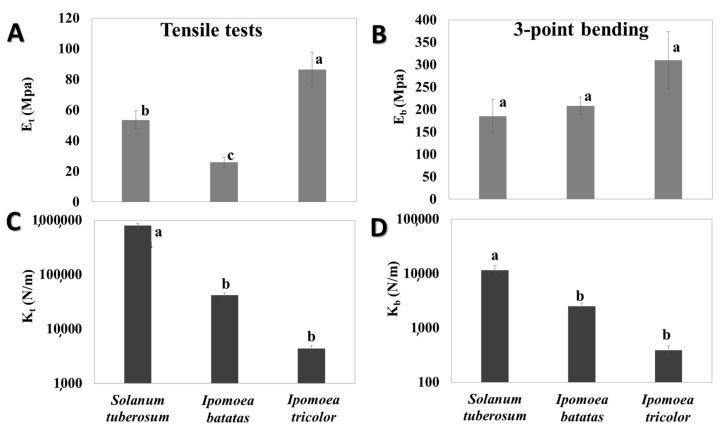
Experimental biomechanical parameters of the stems. (**A**,**C**) Tensile tests, (**B**,**D**) 3-point-bending tests. (**A**) Tensile modulus, *p* ≤ 0.0001, and (**B**) bending modulus, *p* ≤ 0.0808 (**C**) tensile stiffness, *p* ≤ 0.0001, (**D**) bending stiffness, *p* ≤ 0.0001. Calibration length for tensile stiffness is 53 mm. Calibration length for bending stiffness is 30 mm. Different letters above SD bars annotate significant difference according to Tukey-Kramer post-hoc test. Number of samples was *N* = 10 (*I. batatas*), *N* = 10 (*S. tuberosum*), *N* = 6 (*I. tricolor*) for the tensile tests; *N* = 9 (*I. batatas*), *N* = 7 (*S. tuberosum*), *N* = 7 (*I. tricolor*) for the bending tests.

Both tensile and bending tests were conducted to elucidate the biomechanical behavior of various stem types. While the bending tests demonstrate the basic bio-mechanical behavior of the stems in cases of small flexural deflections upon relatively mild loading conditions (e.g., gravity and mild winds), the tensile tests will reflect the stem behavior upon more extreme loadings states (e.g., rapid winds, herbivores) that involves extensive deflections with substantial stem stretching effects [55,56]. Both the tensile and bending stiffness parameters (structural property) and their corresponding tensile and bending moduli parameters (material property) of the stems were experimentally extracted (see materials and methods section); the results are shown in Figure 4. Note that the tensile and bending moduli are viewed as effective characteristics of the stem material that integrates its anatomy (tissue morphology and vacancies) with the micro-mechanical behavior of the cell walls, and are not an attempt to quantify the specific mechanical characteristics of the individual tissues in the stem. Note that the differences in magnitude between the tensile and bending parameters (stiffness and modulus) are expected, since they correspond to the mechanical response at different loading states. As seen in Figure 4C,D both the tensile stiffness and bending stiffness parameters substantially vary (orders of magnitude) between the different types. The differences in the stiffness might originate from either the cross sectional geometry of the stems (morphological characteristic), or their effective modulus (material-level characteristic; i.e., integration of their anatomical structure, cell walls characteristics and chemical relationships), or both. To investigate the effect possibility of substantial variations in the effective modulus between the different types, the tensile modulus and bending modulus of the stems were calculated and the results are shown in Figure 4A,B. As seen in Figure 4A, the tensile moduli of the different stem types are statistically significant. However, the differences are at maximum four-fold between the extreme types (i.e., *I. tricolor* compared to *I. batatas*), which are far below the two- and three-orders of magnitude difference in the tensile stiffness parameter (Figure 4C). Despite the existence of four-fold difference in the tensile modulus between species, these are clearly minor compared to the stiffness differences that compiles in addition to the macrostructural geometry of the stem. The same trend was also observed for the bending parameters. Specifically, while the bending stiffness values are different by two- and three-orders of magnitude between the different species, the differences in the effective bending moduli between the *S. tuberosum* (or the *I. batatas*) and the *I. tricolor* species are at maximum two-fold, and between the *S. tuberosum* and *I. batatas* species, the bending modulus differences were not even statistically significant. 

### 3.3. Cell wall Composition

**Figure 5:**
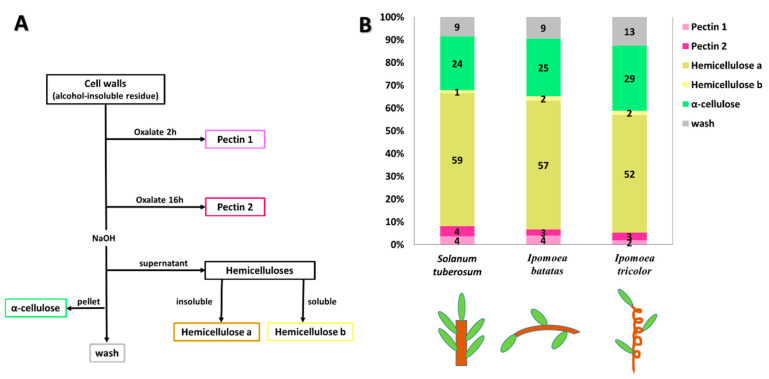
Fractionation of stem cell walls into broad polysaccharide classes. (**A**) fractionation method scheme (after O’Rourke et al., 2015 [54]), (**B**) Comparative cell wall composition in potato, sweet potato and morning glory stems.

In order to compare the cell wall compositions of the three species the cell wall components were fractionated into broad classes, following a method adapted from [54], (Figure 5). Alcohol insoluble residue (AIR) was prepared from the uppermost mature stem internodes. Cell wall components were extracted from the AIR and separated into six fractions: ‘pectin I’, ‘pectin II’, ‘hemicellulose a’, ‘hemicellulose b’, ‘α-cellulose’, ‘wash’ [54], (see Materials and Methods and Figure 5A). Subsequently, the relative proportion of each of the different fractions for each species was determined. All three species were found to possess similar proportions of the different classes of cell wall polymers (Figure 5B). Hemicelluloses were the largest fraction composing ~60% of the cell wall carbohydrates. A further ~25% was constituted by α-cellulose with pectins making up a further 5–8%. In the climber *Ipomoea tricolor* ‘hemicellulose a’ content was a bit lower (52% compared to 59% and 57% in potato and sweet potato respectively), and the α-cellulose content was slightly higher (29% compared to 24% and 25%). 

### 3.4. Immunolocalisation of Specific Cell Wall Epitopes

**Figure 6:**
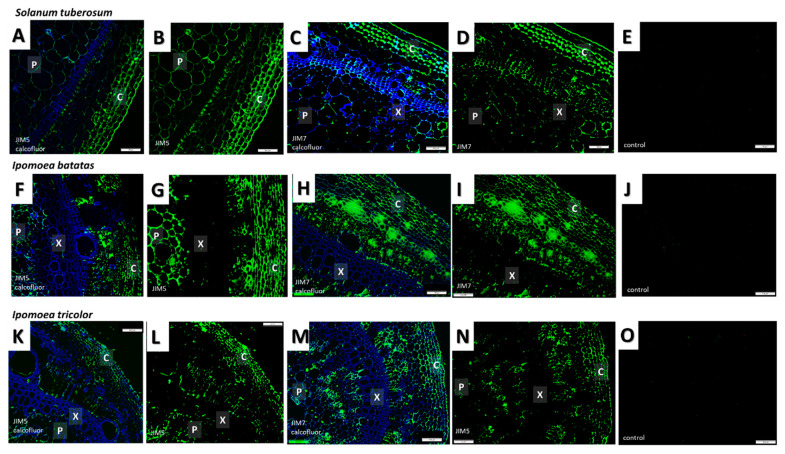
Immunofluorescence detection of epitopes recognised by JIM5 (typically homogalacturonan low levels of esterification) and JIM7 (typically highly esterified homogalacturonan) in *Solanum tuberosum* (**A**–**E**), *Ipomoea batatas* (**F**–**J**) and *Ipomoea tricolor* (**K**–**O**) stems. (**A**,**C**,**F**,**H**,**K**,**M**) Calcofluor white (blue fluorescence) staining of cellulose and is overlaid with (green fluorescence) staining from mAbs. Both JIM7 (**B**,**G**,**L**) and JIM5 (**C**,**I**,**N**) had similar labeling patterns for all three species, being abundant in all the tissues, except the xylem. (**E**,**J**,**O**) negative controls without primary antibody. Abbreviations: X—xylem, C—collenchyma, P—Pith. Scale bars—100 μm. *N* = 3.

**Figure 7:**
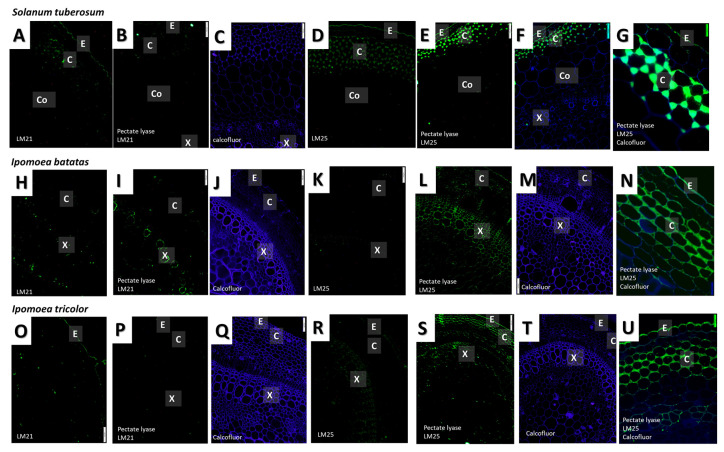
Immunofluorescence labeling of epitopes recognised by LM21 (β-linked mannans) and LM25 (xyloglucans) in *Solanum tuberosum* (**A**–**G**), *Ipomoea batatas* (**H**–**N**) and *Ipomoea tricolor* (**O**–**U**) stems. Calcofluor white staining (blue fluorescence) of cellulose is visible in all cell walls. Abbreviations: X—xylem, C—collenchyma, P—Pith. Scale bars—100 μm. *N* = 3.

Monoclonal antibodies (mAbs) with different epitope affinities were used to label the sections from the different species (Table 2, Figure 6 and Figure 7). Prior to immunolocalization with the mAbs CCRC138 (that recognizes epitopes present in xylans), and LM21 and LM25 (that both recognize epitopes present in xyloglucans), the sections were pretreated with pectate lyase to remove pectins that may hinder binding to hemicelluloses [57]. The immunolabeling patterns for the majority of mAbs used were similar for the three species, with only the labeling patterns observed for CCRC138, LM21, and LM25 showing some variation between the species (Figure 7). 

The sections were labeled with several mAbs known to recognize and bind to epitopes present in pectins including JIM5, JIM7, CCRC-M7, and LM6. JIM5 and JIM7 (which recognize epitopes present in weakly and strongly esterified homogalacturonans, respectively) were found to label the stems of all three species. Labeling was particularly strong in the collenchyma and phloem cell walls, but generally observed throughout the sections, with a similar pattern observed for all three species (Figure 6, Table 2). Labeling with CCRC-M7 (which recognizes epitopes present in rhamnogalacturonans) was most intense in the phloem of all three species. For this mAb the labeling pattern was slightly different in potato compared to the other two species, as labeling was not observed in either the xylem or pith (Table 2). Labeling with the mAb LM6 (which recognizes epitopes present in arabinans) resulted in a similar pattern to that observed for JIM5 and JIM7 with the exception that labeling was additionally detected in the xylem in both *I. batatas* and *I. tricolor* (Table 2).

Sections from the three species were labeled with mAbs CCRC-138, LM21 and LM25 that recognize epitopes present in xylans, mannans, and xyloglucans, respectively. In contrast to labeling with mAbs that recognize epitopes present in pectins, these mAbs exhibited different species-specific labeling patterns. The xylem and phloem of all three species labeled strongly with CCRC-138 (that recognizes epitopes present in xylans). However, the collenchyma of *I. batatas* and *I. tricolor* were also weakly labeled and one *S. tuberosum* section showed very weak fluorescence in the epidermis and collenchyma. Labeling with the mAbs LM25 and LM21 was not observed for sections that were not pre-treated with pectate lyase suggesting that binding to the wall components containing the epitopes that they label (xyloglucans and mannans respectively) was hindered by the presence of pectins. Following pretreatment with pectate lyase, low levels of florescence were observed for LM21 in the epidermis of all three species. LM21 also labeled the collenchyma of *S. tuberosum* and *I. batatas* and the xylem of *I. batatas*. LM25 (which recognizes epitopes present in xyloglucans) strongly labeled the epidermis and collenchyma of all three species, and the xylem, phloem and pith of *I. tricolor* and *I. batatas*. 

The xylem of all three species was labeled with mAb JIM8, which recognizes epitopes present in arabinogalactan proteins (AGPs). The epidermis of *I. batatas* and *I. tricolor* was also weakly labeled (Table 2). 

## 4. Discussion

As climbers have less need for structural self-support, they exhibit traits consistent with resource allocation to growth, rather than mechanical stiffness [58]. Indeed, stem morphology showed substantial changes with the change of habit. Thus, while stems of the climber, morning glory, were significantly the most slender, the self-supporting potato stems had relatively short and thick internodes (Figure 2). A thick stem increases mechanical stiffness. However, slender climbers instead invest material into vertical growth. Prostrate sweet potato (*I. batatas*) and erect potato (*S. tuberosum*) stems were both circular in cross-section, while the stems of the climber morning glory (*I. tricolor*) were more elliptical and slightly flattened (Figure 2). Twining stems from an ascending helix around supports, and to retain their position they generate a squeezing force towards the support [59]. The helical form causes the climbing stem to be very unstable in compression but extraordinarily stable in tension, preventing slipping under gravitational loads [60]. The stability of twining stems under tensional load indicates the importance of friction. A flatter stem allows a better friction between the stem and the support. It is interesting that the self-supporting potato had a wide and circular stem, prostrate sweet potato had a slender and circular stem, and the climber morning glory had a slender elliptic stem. Each stem shape in this case appears to correlate with the plant’s growth habit. 

All three species have a similar anatomy (Figure 3), having predominantly non-lignified stems with peripheral collenchyma serving as the mechanically supporting tissue. The climber *I. tricolor* did not exhibit a special anatomical type of variant growth, as seen in various lianas [61]. However, it is interesting that while in the self-supporting stem of potato the xylem is confined to small vascular bundles, in the other two species the xylem forms a complete circle and is well developed. Also, the potato stem has an empty pith, a common occurrence observed in herbaceous plants. The empty center does not weaken the stem, as the periphery is the load-bearing area, and the periphery in potato is strengthened by thick collenchyma cells. It would be interesting to compare the stem structure of less erect potato varieties with the one used in this study. It is notable that in another Solanaceae, tomato, SP (self-pruning) and DGT (diageotropica) mutations are known to change the plant habit, creating shorter and wider stems, while each gene has an opposite influence on large xylem vessels proportion [62]. Such mutants could serve as a good system for testing biomechanical assumptions. 

Cell wall composition was found to be relatively similar in all three species (Figure 5). Much work has been done on comparative cell wall composition within and between different taxonomic groups, and plants that are closely related phylogenetically tend to have a similar composition [25,26,63]. Since all our plants belong to the Solanales, their global cell wall composition would be expected to be similar [63]. For example members of the Solanales possess xyloglucans with a characteristic composition and structure [64]. However, cell wall composition is also known to vary at the cellular level within a plant. Therefore, we carried out immunolabeling using mAbs to specific cell wall components to identify whether any differences in labeling patterns were observed between the different growth habits. Although immunolabeling suggested that xyloglucans were less abundant in the self-supporting potato stems (Figure 7, Table 2), such a small difference likely does not account for the significant differences in biomechanical function between the species studied [65,66,67]. Similarly, a study of the cell wall composition in two tropical lianas *Bauhinia guianensis* and *Condylocarpon guianense* indicated that their total monosaccharide composition is largely similar to that of woody plants, with the exception that their hemicellulose content was found to be slightly elevated [31]. Even more interestingly, these lianas change their habit from self-supporting to lianescent, with relatively small corresponding changes in cell wall composition such as alterations in cellulose microfibril angle [31]. However, the most striking and consistent ontogenetic changes associated with growth phase related to anatomy [31] and suggest that cell wall composition is not the only driver of biomechanical function.

The importance of geometry rather than material properties is strongly emphasized by the biomechanical measurements, and by various previous studies on plant biomechanics [68,69,70,71,72,73,74,75]. The effective modulus of all stem types, from both the tensile and bending experiments, provided values of the same order of magnitude—which also correspond to those of the liana *Maripa scandens* [76]. This similarity in the effective modulus values between the species indicates that the intrinsic material properties of those species stems are not substantially different. Correspondingly, the underlying structure, i.e., the anatomy and cell wall composition, remained largely unchanged. On the contrary, the corresponding stiffness parameters showed substantially different values, which vary over several orders of magnitudes. These stiffness parameters, which are the actual load-bearing characteristics of the stem that indicate its response to mechanical forces, provide an adequate indicator for its native biomechanical function. Both the tensile and bending stiffness parameters include a multiplication of the effective modulus by a geometrical factor determined by the stem cross section (area in tensile and moment of inertia in bending). Since the effective (tensile or bending) moduli of all stems are at the same orders of magnitudes, but their stiffness parameters are clearly different in their orders of magnitudes, it can be concluded that the geometrical characteristics of the stems, and not their material-level characteristics, are in fact the governing factors on their biomechanical function. In other plants, stem geometrical characteristics, including the local orientation of the stem and diameter, commonly change along the stem to optimize their load-bearing capabilities [77,78]. Similar strategies of geometrical-level (and not material-level) adaptation strategies can be found in other plant organs (e.g., the tapering of foliage leaves toward their connection with the stem). These modifications increase the local moment of inertia and as a consequence reduce the local stresses at that region and prevent mechanical failure [68]. 

Yet, our conclusions may not be universally applicable. *S. tuberosum, I. batatas,* and *I. tricolor* belong to closely related families, and are the products of divergent evolution. *S. tuberosum.*, *I. batatas,* and *I. tricolor* diverged, from a common ancestral lineage, to different structural types, and their differences in growth habit and associated biomechanical properties are determined by morphology. However, climbers have evolved independently many times and in different plant groups, including ferns, gymnosperms, and angiosperms. Moreover, climbers, especially woody lianas, are known to possess numerous structural modifications. Thus, in different taxonomic groups climbers could have found different biomechanical solutions, some of which may be based on cell wall modifications or anatomical changes. At this point many questions remain.

Plant stems are considered to promote designated mechanical properties that support their functional characteristics as load-bearing elements and have a highly efficient functional anatomy—features that have interested scientists for a long time. No less significantly, plant stems supply us with various economically important materials; thus understanding the structural properties of stems is crucial for efficient production and utilization of stem-derived materials.

Understanding the functional properties of different growth forms on different structural and functional levels will enable us a better understanding of material properties of plant stems and is likely to have considerable biomimetic potential.

## Figures and Tables

**Table 1:** List of antibodies used in this study.

Cell Wall Components	mAb	Secondary Antibody	Supplier	Epitope Specificity
Pectins	JIM5	rat	Plantprobes	Predominantly non-methyl-esterified homogalacturonans [46]
	JIM7	rat	Plantprobes	Methyl-esterified homogalacturonans [46]
	CCRC-M7	mouse	Carbosource	Rhamnogalacturonan I (specifically -trimer of beta-(1,6)-Gal) [47]
	LM6	rat	Plantprobes	(1→5)-β-Arabinan [48]
Hemicelluloses	LM21	rat	Plantprobes	Mannans, glucomannans, galactomannans (β-(1→4)-manno-oligosaccharides) [49]
	LM25	rat	Plantprobes	Xyloglucan (XLLG, XXLG and XXXG oligosaccharides of xyloglucan) [50]
	CCRC-M138	mouse	Carbosource	Xylan-6 [51]
AGPs	JIM8	rat	Plantprobes	Arabinogalactan-proteins (AGPs) [52]
Extensins	LM1	rat	Plantprobes	Angiosperm extensins (HRGP) [53]

**Table 2:** Distribution and abundance of cell wall epitopes in the different tissues for three members of the Solanales: *Solanum tuberosum* (self-supporting), *Ipomoea batas* (trailer), *Ipomoea tricolor* (twining climber).

mAb		*Solanum Tuberosum*					*Ipomoea Batatas*					*Ipomoea Tricolor*				
		Epidermis	Collenchyma	Xylem	Phloem	Pith	Epidermis	Collenchyma	Xylem	Phloem	Pith	Epidermis	Collenchyma	Xylem	Phloem	Pith
**Pectin**	***JIM5***	++	+++	-	+++	++ ^1^	++	+++	- ^2^	+++	++	++	+++	- ^2^	+++	++ ^1^
	***JIM7***	+++	+++	++	+++	++	+++	+++	++	+++	++	+++	+++	+-	+++	++
	***CCRC-M7***	+	+- ^1^	- ^3^	+++	-	++	++ ^1,4^	+	+++	+	++	++ ^4^	+	+++	+
	**LM6**	++	+++	-	++	++	++ ^5^	+++	++ ^6^	++	++	++ ^5^	+++	++ ^6^	++	++
**Hemicellulose**	**CCRC-M138**	- ^3^	- ^3^	+++ ^7,8^	+++ ^9,10^	-	-	+	+++ ^2,7,8^	+++ ^9^	-	-	+-	+++ ^2,7,8^	+++ ^9^	-
	**LM25 unmasked**	++	+++	-	-	-	++	+++	++	++	++	++	+++	++	++	++
	***LM21* unmasked**	+-	+	-	-	-	+-	+-	+ ^7^	-	-	+-	-	-	-	-
**AGP**	**JIM8**	-	-	++ ^8^	-	-	+	-	++ ^8^	-	-	+	-	+ ^8^	-	- ^3^
**Extensins**	**LM1**	- ^3^	-	-	-	-	-	-	-	-	-	-	-	-	-	-

-None, +- Trace, + Weak, ++ Intermediate, +++ Strong. ^1^ Especially cell corners. ^2^ Weakly in large cavities. ^3^ Weak in one sample, probably autofluorescence. ^4^ Inside the cells. ^5^ Very strong in stomatal complex. ^6^ In xylem rays. ^7^ In xylem vessels. ^8^ In xylem parenchyma. ^9^ Outer phloem. ^10^ Inner phloem.

## Supplementary Materials

The following are available online at https://www.mdpi.com/2223-7747/9/6/678/s1, Figure S1: Lignin stain in *Solanum*, *I. batatas* and *I. tricolor*. Phloroglucinol-HCl stain of lignin according to Shtein et al. 2017 [74]. Note that only xylem is stained, Figure S2: Variations and sensitivity in the normalized bending modulus with the sample SDR.
