# Supplementary material for: Solanales Stem Biomechanical Properties Are Primarily Determined by Morphology Rather Than Internal Structural Anatomy and Cell Wall Composition
Source: Plants (Basel). 2020 May 27;9(6):678. doi: 10.3390/plants9060678 (PMC7356250; doi:10.3390/plants9060678)
Supplement: Supplementary file 1 [file plants-09-00678-s001.pdf]

Supporting Information

Solanales stem biomechanical properties are primarily determined by morphology rather than internal structural anatomy and cell wall composition

Ilana Shtein[1,2,4], Alex Koyfman[1,3], Amnon Schwartz[2], Zoë A. Popper[4], Benny Bar-On[1*]

S1. Lignin stain

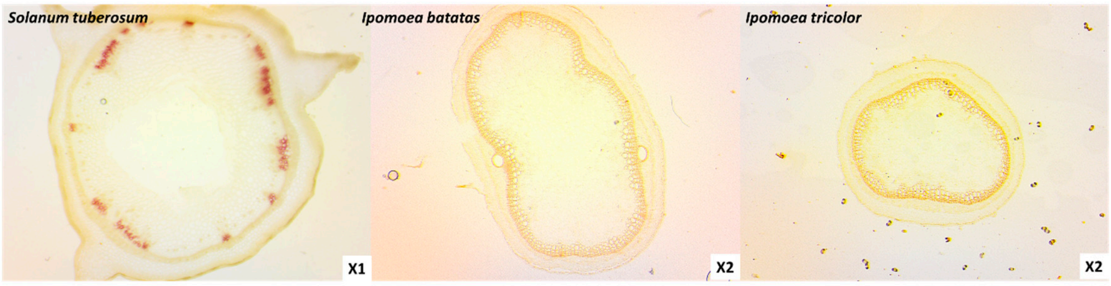

Figure S1. - Lignin stain in *Solanum*, *I. batatas* and *I. tricolor*. Phloroglucinol-HCl stain of lignin according to Shtein et al. 2017b. Note that only xylem is stained.

S2. The effect of the SDR on the bending modulus

To verify absence of shear effects in our 3-point-bending experiments Figure S1.A plots the bending modulus (E_B) of each sample, calculated via (Eq. (4) in the main text, along with their corresponding span-to-depth (SDR) values. Additionally, the local sensitivity of the bending modulus (of each species), indicated by the gradient $d(E_B)/d(SDR)$ and calculated via the finite difference in Eq. S1.1, are plotted in Figure S2.B.

$$\frac{d(E_B)}{d(SDR)} = \frac{E_B(i+1) - E_B(i)}{SDR(i+1) - SDR(i)} \quad ; \quad SDR(i+1) > SDR(i) \qquad \text{(S1.1)}$$

In 3-point-bending experiments that involve non-negligible shear effects, E_B is expected to monotonically increase with the sample SDR, along with a persistent positive sensitivity to local variations in the SDR value (e.g., Spatz and Vincent, (1994)). As clearly shown in Figure S1 neither of these effects is observed in the present study, which indicates that the contributions of shear effects are indeed negligible.

<u>Reference</u>

Spatz, H. C., & Vincent, J. F. V. (1996). Young's moduli and shear moduli in cortical bone. *Proceedings of the Royal Society of London. Series B: Biological Sciences*, *263*(1368), 287-294.

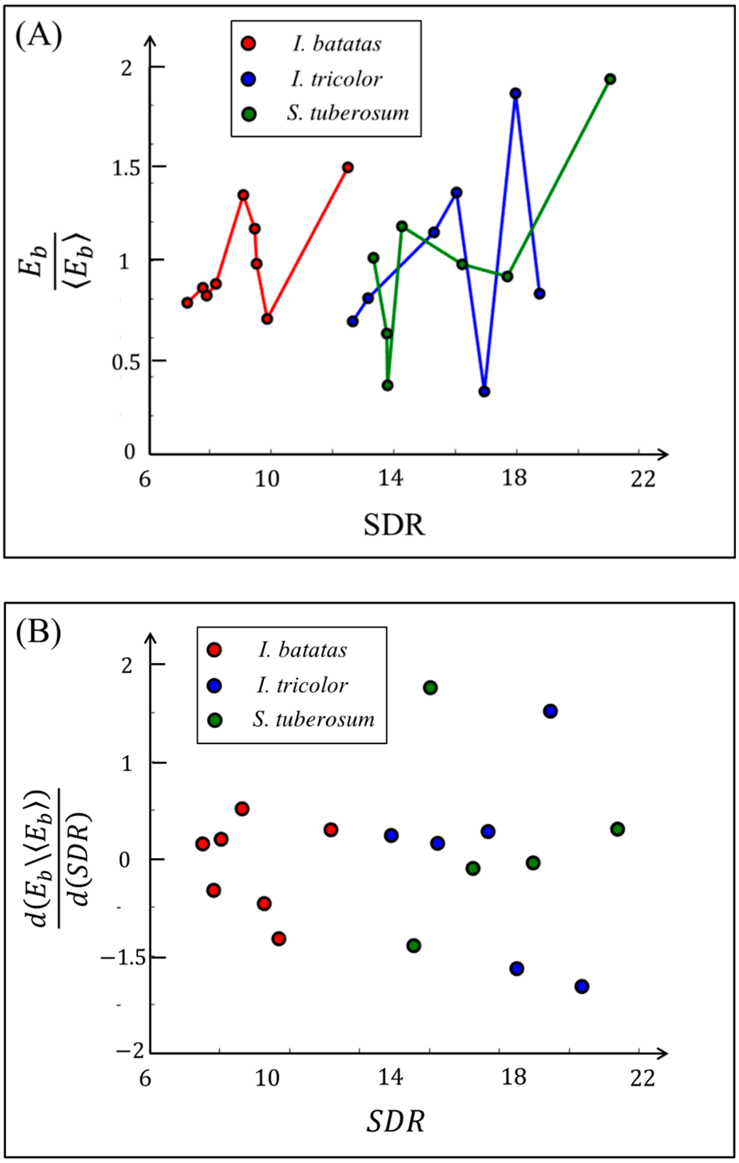

Figure S2. (A) Variations in the normalized bending modulus with the sample SDR in the present 3-point-bending experiments. The lines connect data-points with successive SDR values. Each bending modulus was normalized by the mean bending modulus of the corresponding species. (B) Local sensitivity of the normalized bending modulus, calculated via eq. (S1.1).
